# Integration of datasets for individual prediction of DNA methylation-based biomarkers

**DOI:** 10.1186/s13059-023-03114-5

**Published:** 2023-12-05

**Authors:** Charlotte Merzbacher, Barry Ryan, Thibaut Goldsborough, Robert F. Hillary, Archie Campbell, Lee Murphy, Andrew M. McIntosh, David Liewald, Sarah E. Harris, Allan F. McRae, Simon R. Cox, Timothy I. Cannings, Catalina A. Vallejos, Daniel L. McCartney, Riccardo E. Marioni

**Affiliations:** 1 School of Informatics, University of Edinburgh, Edinburgh, EH8 9AB UK; 2 Centre for Genomic and Experimental Medicine, Institute of Genetics and Cancer, University of Edinburgh, Edinburgh, EH4 2XU UK; 3 Edinburgh Clinical Research Facility, University of Edinburgh, Edinburgh, EH4 2XU UK; 4 Division of Psychiatry, Centre for Clinical Brain Sciences, University of Edinburgh, Edinburgh, UK; 5 Department of Psychology, Lothian Birth Cohorts, University of Edinburgh, Edinburgh, EH8 9JZ UK; 6 Institute for Molecular Bioscience, University of Queensland, Brisbane, Australia; 7 Maxwell Institute for Mathematical Sciences, School of Mathematics, University of Edinburgh, Edinburgh, EH9 3FD UK; 8 MRC Human Genetics Unit, Institute of Genetics and Cancer, University of Edinburgh, Edinburgh, EH4 2XU UK; 9 The Alan Turing Institute, London, UK

**Keywords:** DNA methylation, Prediction, Biomarker

## Abstract

**Background:**

Epigenetic scores (EpiScores) can provide biomarkers of lifestyle and disease risk. Projecting new datasets onto a reference panel is challenging due to separation of technical and biological variation with array data. Normalisation can standardise data distributions but may also remove population-level biological variation.

**Results:**

We compare two birth cohorts (Lothian Birth Cohorts of 1921 and 1936 — n_LBC1921_ = 387 and n_LBC1936_ = 498) with blood-based DNA methylation assessed at the same chronological age (79 years) and processed in the same lab but in different years and experimental batches. We examine the effect of 16 normalisation methods on a novel BMI EpiScore (trained in an external cohort, *n* = 18,413), and Horvath’s pan-tissue DNA methylation age, when the cohorts are normalised separately and together. The BMI EpiScore explains a maximum variance of *R*^2^=24.5% in BMI in LBC1936 (SWAN normalisation). Although there are cross-cohort *R*^2^ differences, the normalisation method makes a minimal difference to within-cohort estimates. Conversely, a range of absolute differences are seen for individual-level EpiScore estimates for BMI and age when cohorts are normalised separately versus together. While within-array methods result in identical EpiScores whether a cohort is normalised on its own or together with the second dataset, a range of differences is observed for between-array methods.

**Conclusions:**

Normalisation methods returning similar EpiScores, whether cohorts are analysed separately or together, will minimise technical variation when projecting new data onto a reference panel. These methods are important for cases where raw data is unavailable and joint normalisation of cohorts is computationally expensive.

**Supplementary Information:**

The online version contains supplementary material available at 10.1186/s13059-023-03114-5.

## Background

There is an increasing focus on the application of epigenetic biomarkers in large cohort studies for health research [1]. For example, DNA methylation (DNAm)-based predictors — epigenetic scores or EpiScores — of adiposity, smoking, alcohol consumption (traits which typically suffer from measurement error due to recall bias), and protein levels may help to stratify individuals into risk groups and predict disease outcomes [2, 3].

However, the DNAm data sourced from different populations and lab environments can have a technical and biological variation that is difficult to partition. Normalising DNAm datasets can account for technical variation but may remove meaningful biological variation. Understanding the impact of normalisation is vital if future studies are to integrate multiple methylation datasets. Recent work has also described heterogeneity when applying different normalisation methods to replicate samples from the same individual [4]. That study showed considerable variation in the normalisation pipeline that yielded the highest intraclass correlations for 41 different EpiScores.

The Illumina Infinium HumanMethylation450 and EPIC arrays assess methylation genome-wide [5, 6] and are widely used by cohort studies. Following a whole-genome amplification step, probes hybridise to target CpG sites and fluorescent markers signal methylation status. The arrays measure methylation using two probe types (Type I and Type II). Type I probes have two 50-mer probes for each CpG site, one of which hybridises to the methylated site (M) and the other to the unmethylated site (U). Type II probes are a single probe with two different dye colours to differentiate between M and U states.

Quantile normalisation (QN) is a nonlinear transformation that ensures the array-wide distributions of CpG values are identical by replacing the raw CpG values with the mean of all CpG features with the same rank [7]. QN can be used to correct bias due to differences between methylated and unmethylated dye intensities (dye-bias correction) and bias due to Type I and Type II probe differences (between-array normalisation). In addition, background adjustment can control for the offset between Type I and Type II probe intensities. In addition to QN, which utilises information across samples, within-array (i.e. sample-indepedent) approaches also exist. These approaches include subset within-array normalisation (SWAN), which reduces the difference in distributions between Type I and Type II probes, based on a random subset of biologically similar probes [8]; beta-mixture quantile (BMIQ) normalisation, which performs an adjustment on Type II probes, transforming their distribution to one more similar to Type I probes [9]; peak-based correction (PBC), a correction method which rescales Type II probe distributions on the basis of Type I probe data [10]; and normal-exponential out-of-band (Noob) normalisation, which performs background correction and dye-bias equalisation [11].

If technical noise across different datasets can be accounted for, new samples or datasets can be normalised individually rather than re-normalising all data together, which is computationally expensive. Here, we apply various quantile normalisation methods to two independent cohorts of age-matched older adults (considered separately and jointly) to determine which approach performs best for the projection of a new set of individuals onto a reference dataset.

## Results

**Fig. 1:**
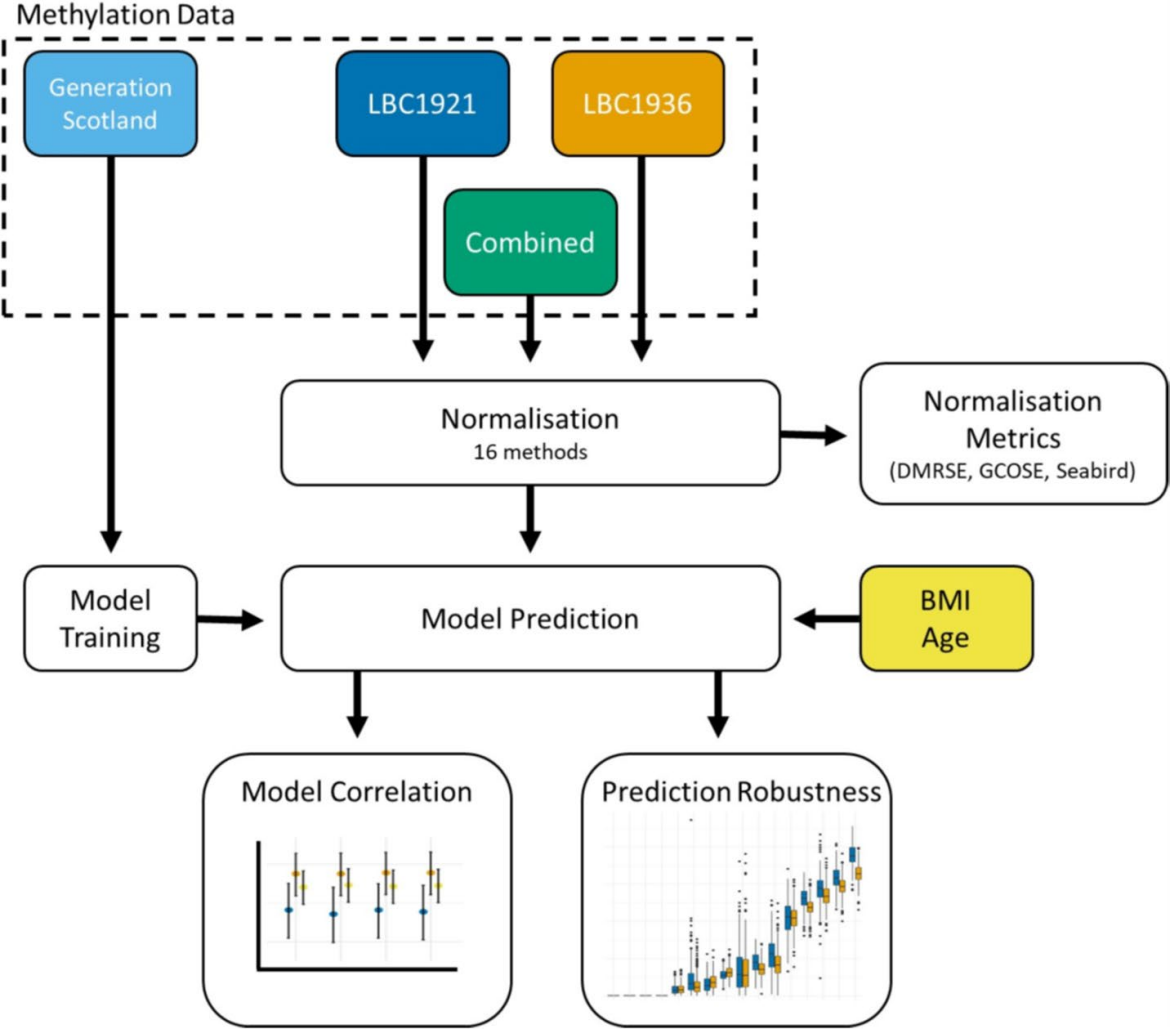
Schematic of normalisation pipeline, model training, and prediction steps

Sixteen normalisation approaches [7–13] were ranked amongst three datasets (the Lothian Birth Cohort of 1921 (LBC1921), the Lothian Birth Cohort of 1936 (LBC1936), and LBC1921 + LBC1936 combined; Fig. 1) [14]. Methylation was assessed on the Illumina 450k array in two separate experiments. First, samples from 387 individuals from LBC1921 taken between 1999 and 2001 at a mean age of 79.1 (SD = 0.58) years were processed. Second, samples from 498 individuals from LBC1936 taken between 2014 and 2017 at a mean age of 79.3 (SD = 0.62) years were processed. The combined cohort contained 885 individuals. Pre-normalisation filtering steps are described in the Methods.

While no single method consistently ranked highest under the three metrics considered (DMRSE, GCOSE, seabird — ‘Methods’), daten2 and naten performed well overall (Additional file 1: Fig S1–S2). Poorer performances were observed for subset quantile normalisation (Tost method), PBC and unnormalised (raw) data, based on the three wateRmelon metrics.

### DNAm-based BMI Prediction by normalisation method

**Fig. 2:**
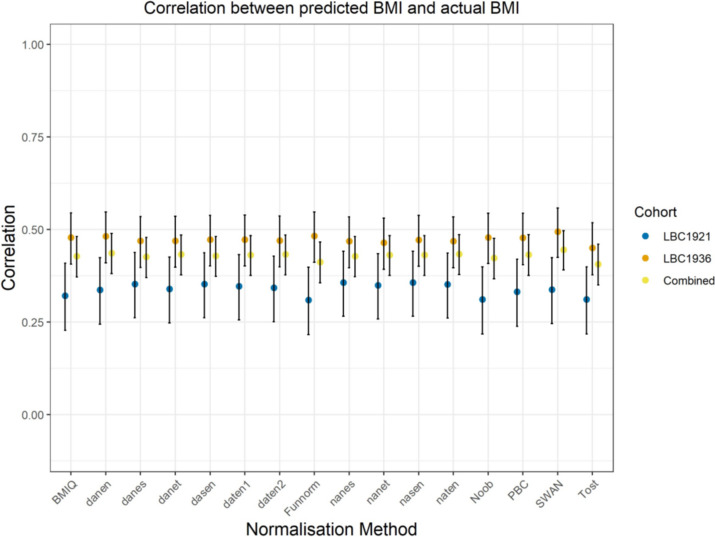
Pearson correlations between observed BMI and the BMI EpiScore across normalisation methods. Error bars represent the 95% confidence intervals

Pearson correlations between observed BMI and a BMI epigenetic score (EpiScore; trained in an independent cohort of 18,413 individuals using elastic net regression — ‘Methods’) are shown in Fig. 2 and Additional file 2: Table S1 for all normalisation methods. The mean correlations were 0.34 (SD 0.02) for LBC1921 and 0.47 (SD 0.01) for LBC1936 — the maximum correlation was 0.49 (incremental *R*^2^ = 24.5% when comparing linear regression models of log(BMI) against age and sex with/out a BMI EpiScore) for the SWAN normalisation method in LBC1936.

### Prediction robustness metrics assessment for BMI

**Fig. 3:**
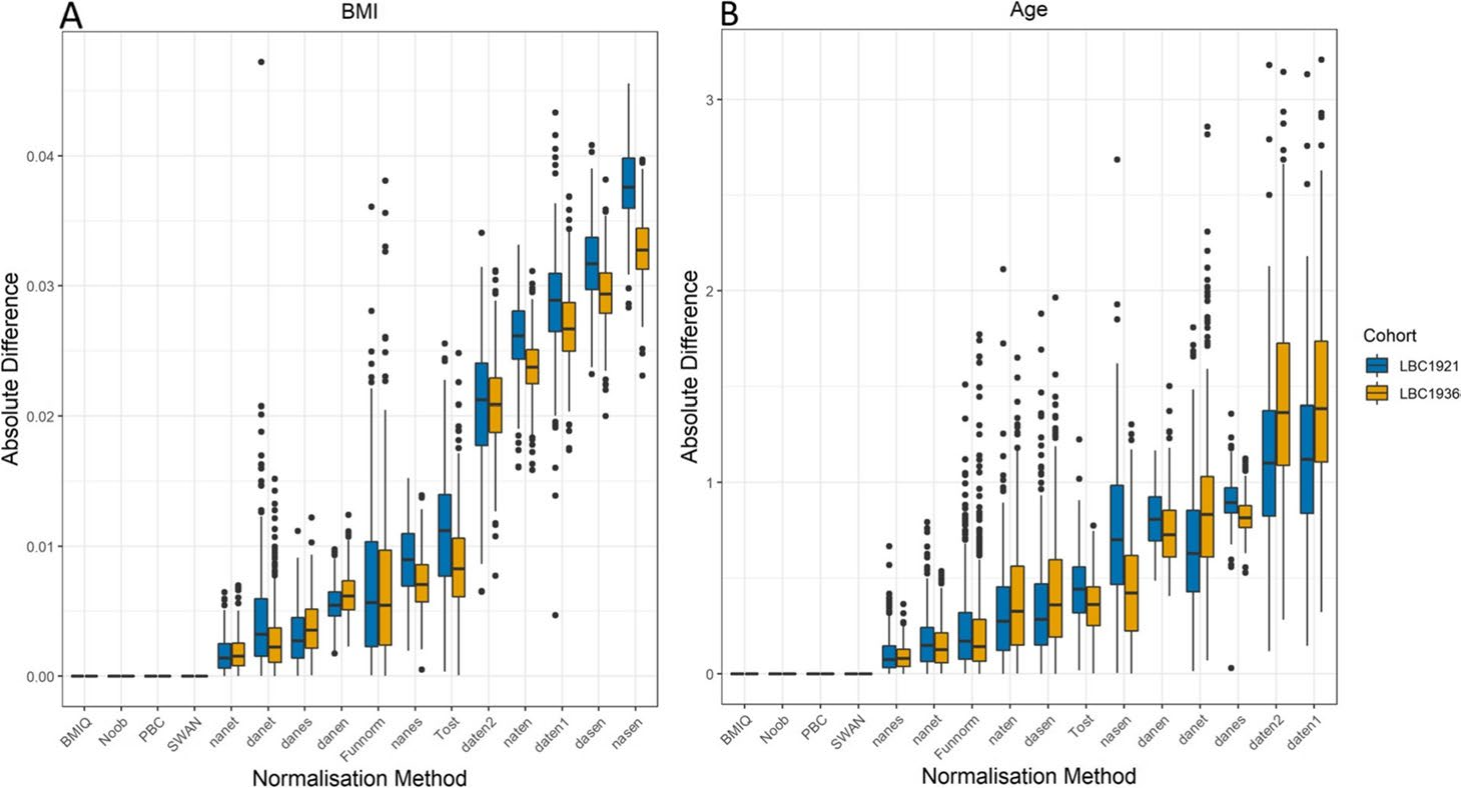
Box plots comparing median absolute value difference in BMI EpiScores and age EpiScores between separately and jointly normalised cohorts

**Fig. 4:**
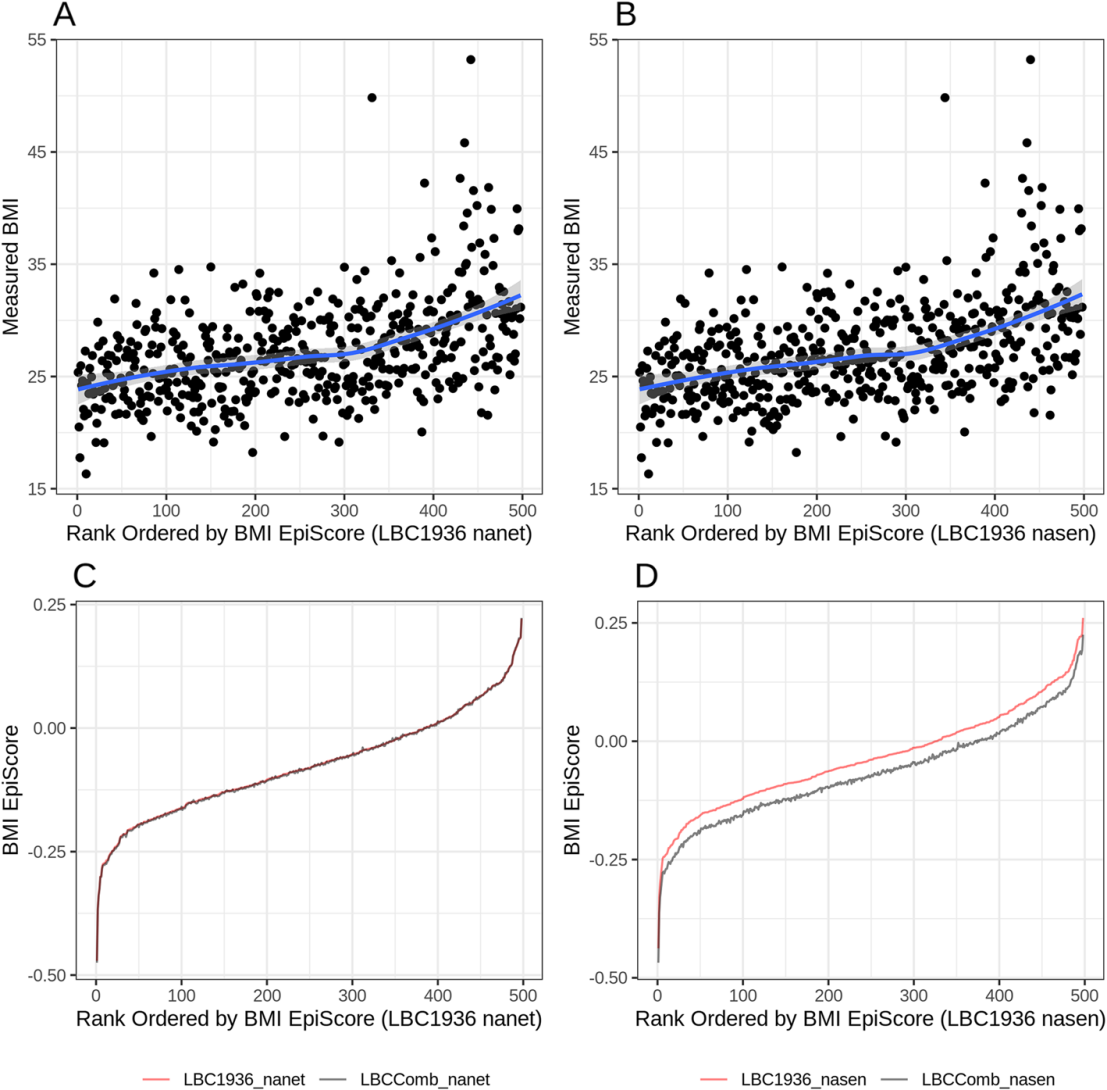
Measured BMI and BMI EpiScores in LBC1936 for the nanet (lowest MAD) and nasen (highest MAD) normalisation methods after normalising the LBC1936 data on its own and then jointly with LBC1921. Actual BMI (kg/m^2^) is plotted for individuals in ascending order of their LBC1936 EpiScore values (panels **A**–**B**). BMI EpiScores are plotted against individuals in ascending order of their EpiScore values for the LBC1936 normalised on its own (LBC1936 - red line) and together with LBC1921 (LBCComb - black line; panels **C**–**D**)

While the choice of normalisation method had little effect on the EpiScore correlations with actual BMI, differences amongst the normalisation methods were present when looking into deviations in EpiScore predictions for individuals. Figure 3 shows the median absolute difference in EpiScores for each normalisation method. For all EpiScore predictions, the within-array methods (SWAN, Noob, PBC and BMIQ) had no median difference because they are entirely sample-wise methods (i.e. normalisation is specific to each sample). Of the other methods, nanet was the best performing with the lowest median absolute difference in the predicted BMI EpiScores (0.0014 and 0.0015 units of log(BMI) adjusted for age, sex, and genetic PCs in LBC1921 and LBC1936, respectively — approximately 0.01 kg/m^2^ after de-scaling, Additional File 1: Appendix 1) for LBC1921 participants when normalised separately and together with LBC1936. Figure 4 highlights the similarity in EpiScores for the nanet-normalised data compared to nasen (the between-array methods with the smallest and largest median absolute differences, respectively) for BMI in LBC1936.

### Prediction and robustness for epigenetic age

To explore if the findings varied by the EpiScore being tested, we re-ran the analyses for Horvath’s 2013 predictor of chronological age [15]. Whereas epigenetic clocks typically show correlations above 0.9 in populations with wide age ranges, this metric is less sensitive within narrow age ranges (e.g. birth cohorts). Here, we observed correlations between 0.004 and 0.15 across the cohorts and normalisation methods. We also observed a different rank ordering of the median errors for the normalisation approaches (Fig. 3). The nanet method performed consistently well across predicted age and BMI, whereas the daten1 method showed consistently poor performance across traits. Mixed performance was observed for other methods (e.g. danet).

## Discussion

The removal of technical noise from DNAm datasets is essential for more reliable projections of new individuals onto a reference dataset [16]. Optimal normalisation methods should give similar predictions irrespective of whether a dataset has been normalised independently or jointly with a potential reference dataset.

Here, we showed that, for the generation of BMI EpiScores, between-array methods such as nanet and danet yield similar predictions irrespective of whether the input data was normalised with the reference dataset. As expected, within-array methods showed no differences in predictions between datasets. A range of correlations was observed between phenotypic BMI and the BMI EpiScores using these methods, with SWAN performing well in both cohorts. While within-array methods result in a zero mean absolute difference, they also guarantee that no batch-level effects are taken into account. Specifically, they do not attempt to minimise systematic errors that may have taken place during data collection and processing. It is therefore possibly a chance finding (or a result of relatively homogeneous input data) that SWAN yielded the best BMI EpiScore correlation with measured BMI. Perhaps reflecting this, another within-array method, Noob yielded the second- and third-lowest correlation between BMI and the EpiScore in LBC1921 and the jointly normalised cohorts, respectively. In contrast, nanet and danet gave both high correlations and low median absolute differences. A common feature between these methods is the application of dye-bias correction to Type I and Type II probes together.

When projecting BMI EpiScores for a new cohort, between-array normalisation methods that don’t perform dye-bias correction (e.g., nasen, dasen, naten) gave greater differences in EpiScores when the new cohort was normalised on its own versus jointly with the second, reference population. Whether Type I or Type II probes were normalised separately or together did not seem to have a clear effect on EpiScore robustness. Technical noise due to dye-bias was therefore the main cause of discrepancies for the BMI EpiScores robustness.

While no single best normalisation method is clearly highlighted, we identified strengths of normalisation techniques that will generalise well to a new dataset. Methods that do not correct for dye-bias appear to have limited utility. This finding is important for the potential deployment of DNAm prediction tools in the healthcare community. Ideally, studies would not have to re-normalise a dataset every time new volunteers or patients are entered, which is both time-consuming and computationally expensive. Quantifying an acceptable median absolute difference between EpiScores generated from different normalisation approaches is likely to vary by both the EpiScore and its application. What might be more important is the preservation of rank order e.g., if this is used to help select individuals for a clinical trial. Ideally, a new individual wouldn’t change their percentile place in a population distribution if their data are normalised separately or together with the reference dataset.

The maximum effect size observed here (*R*^2^ = 24.5%) is larger than the previous best estimate (19.5%) trained in a smaller subset of Generation Scotland and tested in the same LBC1936 cohort [17]. This is a much larger effect size than we observed from a BMI polygenic risk score (*R*^2^ = 10.1%) [2] and is also comparable to the largest effect sizes previously observed for 109 protein EpiScores (see Fig. 2 from [3]). Chronological age is the most widely reported trait with large EpiScore correlations (i.e. Epigenetic Clocks). However, while these clocks show high correlations across populations with wide-age ranges, they perform much less well in birth cohorts or studies with a narrow age range. Applying Horvath’s 2013 predictor [15] to our current datasets yielded a different order of ‘best’ normalisation methods, though nanet performed well across both traits (BMI and age). Considering both the trait as well as the normalisation method is therefore likely to be of importance when combining datasets.

There are also some general limitations around prediction that need to be considered. Firstly, the BMI EpiScore was trained and tested in Scottish populations. Its correlation with observed BMI differed substantially across the two LBC studies, despite minimal heterogeneity in age and background. We hypothesise that these differences may be a result of selection or survival bias. LBC1921 participants were required to be healthy upon recruitment at age 79 compared to LBC1936 volunteers, who were healthy upon recruitment at age 70 but had to survive until the fourth study wave (at age 79) to be considered in these analyses. Differences are also likely to exist when EpiScores are applied to more diverse populations (e.g., wider age ranges, different social backgrounds and ancestries), though the tightly-matched age range across both test cohorts reported here is valuable in that cross-cohort comparisons were not confounded by age differences. The controlled environment in which samples were collected (in a clinic, according to a protocol) and processed (in the same research laboratory) may also be a limitation in that much of the heterogeneity associated with data collection and processing of clinical samples will not be present in our data. Therefore, prior to establishing a ‘best practice’ model, further testing in diverse samples is required. EpiScore predictors for more complex traits or disease risk scores could also be trained [2]. Furthermore, we focused on population average differences across the normalisation strategies, as opposed to variation at the individual level. By definition, if we were to consider new individuals one at a time, then only within array normalisation methods would be applicable. When projecting new DNAm data into an existing dataset, it is likely that any new samples will be analysed in experimental batches due to the costs and time associated with generating the data. Further research should investigate the potential consequences of different normalisation strategies when incorporating data from individual samples into existing datasets

## Conclusions

Most existing EpiScore analyses have focused on relative differences within a cohort or analysis batch [1]. By applying and comparing a variety of normalisation approaches, we suggest individuals or cohorts could be reliably projected onto a reference panel. This will enable users to generate methylation-based scores and risk percentiles for a variety of traits and diseases.

## Methods

### Lothian Birth Cohorts (LBC) of 1921 and 1936 — DNAm quality control

DNAm data was assessed using the Illumina 450k array for 499 individuals from the LBC1936 and 436 individuals from the LBC1921 [14]. Prior to normalisation, each sample went through a number of filtering checks. Probes predicted to cross-hybridise or target a site containing a polymorphism (*n* = 54,192) were removed [18]. *P*-values to quantify signal reliability (detection *P*-values) were computed for each CpG probe. Probes which had more than 1% of samples with a *P*-value greater than 0.05 were removed (7366 LBC1921 probes were removed, 1495 LBC1936 probes were removed) Individual samples where more than 1% of probes had a detection *P*-value greater than 0.05 were removed (49 LBC1921 samples, 1 LBC1936 sample removed) Finally, we removed probes with a bead count of less than three in more than 5% of samples (191 LBC1921 probes removed, 362 LBC1936 probes removed). Following quality control, there were 443,339 remaining probes common across all datasets. There were 387 individuals remaining in the LBC1921 cohort and 498 individuals remaining in the LBC1936 cohort. The combined cohort had 885 individuals.

### Normalisation methods

Sixteen normalisation methods from the Minfi and WateRmelon packages [7–13] were applied to LBC1921, LBC1936, and the combined LBC dataset with the pipeline depicted in Fig. 1.

WateRmelon is an R package that implements several QN methods with systematic nomenclature described in [7]. Methods which start with a ‘d’ apply background adjustment (‘n’ indicates no adjustment). The third letter specifies whether between-array normalisation was performed to Type I and II probes separately (‘s’), together (‘t’), or not at all (‘n’). The final letter indicates whether the dye-bias correction was applied to Type I and II probes separately (‘s’), together (‘t’), or not at all (‘n’). A description of the difference between normalisation methods is shown in Additional file 2: Table S2.

Minfi is an R package [19] that implements three additional normalisation techniques, Noob, Funnorm and Subset-quantile within array normalisation (SWAN). Normal-exponential out-of-bound (Noob) is a within-sample background correction method with dye-bias normalisation for DNAm arrays [11]. Noob uses a normal-exponential convolution method to estimate background distributions by measuring non-specific fluorescence based on out-of-band Type I (i.e. probes in the opposite colour channel - Cy3 vs Cy5). Funnorm, a between-sample normalisation method, makes use of 848 internal control probes and out-of-band probes on the Illumina array to estimate 42 summary measures to account for technical variation [12]. The first two principal components of these summary measures are then used as covariates for intensity adjustment. SWAN consists of two steps [8]. The first step takes a subset of probes, defined to be biologically similar based on CpG content, and determines an average quantile distribution from this subset. The second step adjusts the intensities of the remaining probes by linear interpolation onto the distribution of the subset probes.

In addition to the wateRmelon- and Minfi-implemented functions, we applied 3 widely-cited methods in our comparison: BMIQ, peak-based correction and subset quantile normalisation [9, 10, 13]. BMIQ (a within-array method) first fits a 3-state beta mixture model (0%. 50% and 100% methylation) for Type I and Type II probes separately, in which probes are assigned to the state with maximum probability. This is followed by the normalisation of Type II probes to the distributions of Type I probes in the same group. Peak-based correction independently estimates M-value peaks for Type I and Type II probes, followed by rescaling of the Type II assays to match the estimates obtained for Type I assays. Subset quantile normalisation (Tost method), normalises signal from Type II assays based on a set of Type I ‘anchor’ probes, which are considered to be more reliable and stable.

### Normalisation assessment metrics

Three previously published performance metrics were considered [7]. Differentially Methylated Regions Standard Error (DMRSE) measures the variation at sites defined as uniparentally methylated regions with an expected β value of 0.5. The standard error is computed by dividing the standard deviation of differentially methylated region β values by the square root of the number of samples. Genotype Combined Standard Error (GCOSE) examines highly polymorphic SNPs which have three genotypes: heterozygous or homozygous with the major or minor allele. This metric clusters observations into the three groups based on genotype and computes a mean-squared error for each cluster, then averages the three means. Finally, the Seabird metric computes the area under the curve (AUC) for a predictor trained on sex differences on the X chromosome, of which one is hypermethylated in females. Each of the normalisation metrics was ranked on each of the three metrics; the ranks were then averaged to compute a mean overall rank.

### DNAm predictor of BMI

A DNAm predictor of body mass index (BMI) was derived using elastic net penalised regression (*α* = 0.5) on 18,413 participants from the Generation Scotland study [20]. The lambda value that minimised the mean error in a 10-fold cross-validation analysis resulted in a weighted linear predictor containing 3506 CpGs (see Additional file 3: Table S3). As the Generation Scotland DNAm resource was generated using the EPIC array, CpGs were the first subset to the 445,962 sites that were common to the 450k array and that passed QC in the LBC analyses. They were further pruned to the 200,000 most variable CpG features (ranked by standard deviation) to avoid a memory allocation error in the elastic net model. R’s biglasso package was used to implement an elastic net regression model [21–23]. The input to the model was a 200,000 × 18,413 matrix containing the CpG *M*-values for each individual. The target variable was the residuals from a linear regression model of log(BMI) adjusted for age, sex and 10 genetic principal components. The distribution of BMI in the two LBC studies and Generation Scotland are presented in Additional file 1: Fig S3.

### Prediction and robustness

Predictions of BMI were performed on both LBC datasets and the combined LBC dataset. An individual’s BMI was predicted by weighting their CpG values by the CpG weights from the Generation Scotland elastic net model. Overall model prediction performance was evaluated by Pearson’s correlation coefficient.

Prediction robustness measures a normalisation method’s invariance to datasets being normalised independently, or jointly with another dataset. Robustness was calculated as the median absolute difference between the independent and joint predictions across all individuals. The goal is to identify how the test datasets behave when predictions are made using data normalised jointly or separately. Small median differences indicate normalisation methods that provide similar outputs irrespective of the data being normalised separately or together. Normalisation methods with large median absolute differences result in inconsistent predictions depending on whether new individuals are normalised jointly with previous data or not.

### Supplementary Information

**Additional file 1:** **Fig S1.** Heatmaps of normalisation method ranks for the DMRSE, GCOSE and Seabird metrics. **Fig S2.** Individual scores for the DMRSE, GCOSE and Seabird metrics across normalisation methods applied to LBC1921, LBC1936 and both cohorts combined. **Fig S3.** Density plot of BMI (kg/m^2^) in the Lothian Birth Cohort 1921, the Lothian Birth Cohort 1936, and Generation Scotland. **Appendix 1.** Converting BMI EpiScores back to the original (BMI kg/m^2^) scale.

**Additional file 2:** **Table S1.** Correlations between measured BMI vs BMI EpiScore and age vs age EpiScore in LBC1921, LBC1936 and LBC1921 + LBC1936 (Combined) for every normalisation method. **Table S2.** WateRmelon normalisation method descriptions.

**Additional file 3:** **Table S3.** BMI EpiScore CpGs and weights.

**Additional file 4:** Review history.

## Data Availability

According to the terms of consent for Generation Scotland (GS) participants, access to data must be reviewed by the GS Access Committee. Applications should be made to access@generationscotland.org. Lothian Birth Cohort data are available on request from the Lothian Birth Cohort Study, University of Edinburgh (https://www.ed.ac.uk/lothian-birth-cohorts/data-access-collaboration). Lothian Birth Cohort data are not publicly available due to them containing information that could compromise participant consent and confidentiality. All code is available with open access under the terms of the MIT license at the following GitHub repository: https://github.com/marioni-group/DNAm_Projections_2023 [24]. This code has also been deposited in Zenodo, under DOI 10.5281/zenodo.10096139 [25].
