# Supplementary material for: Integration of datasets for individual prediction of DNA methylation-based biomarkers
Source: Genome Biol. 2023 Dec 5;24:278. doi: 10.1186/s13059-023-03114-5 (PMC10696831; doi:10.1186/s13059-023-03114-5)
Supplement: Supplementary file 1 — Additional file 1: Fig S1. Heatmaps of normalisation method ranks for the DMRSE, GCOSE and Seabird metrics. Fig S2. Individual scores for the DMRSE, GCOSE and Seabird metrics across normalisation methods applied to LBC1921, LBC1936 and both cohorts combined. Fig S3. Density plot of BMI (kg/m2) in the Lothian Birth Cohort 1921, the Lothian Birth Cohort 1936, and Generation Scotland. Appendix 1. Converting BMI EpiScores back to the original (BMI kg/m2) scale. [file 13059_2023_3114_MOESM1_ESM.docx]

**Fig S1:** Heatmaps of normalisation method ranks for the DMRSE, GCOSE and Seabird metrics.


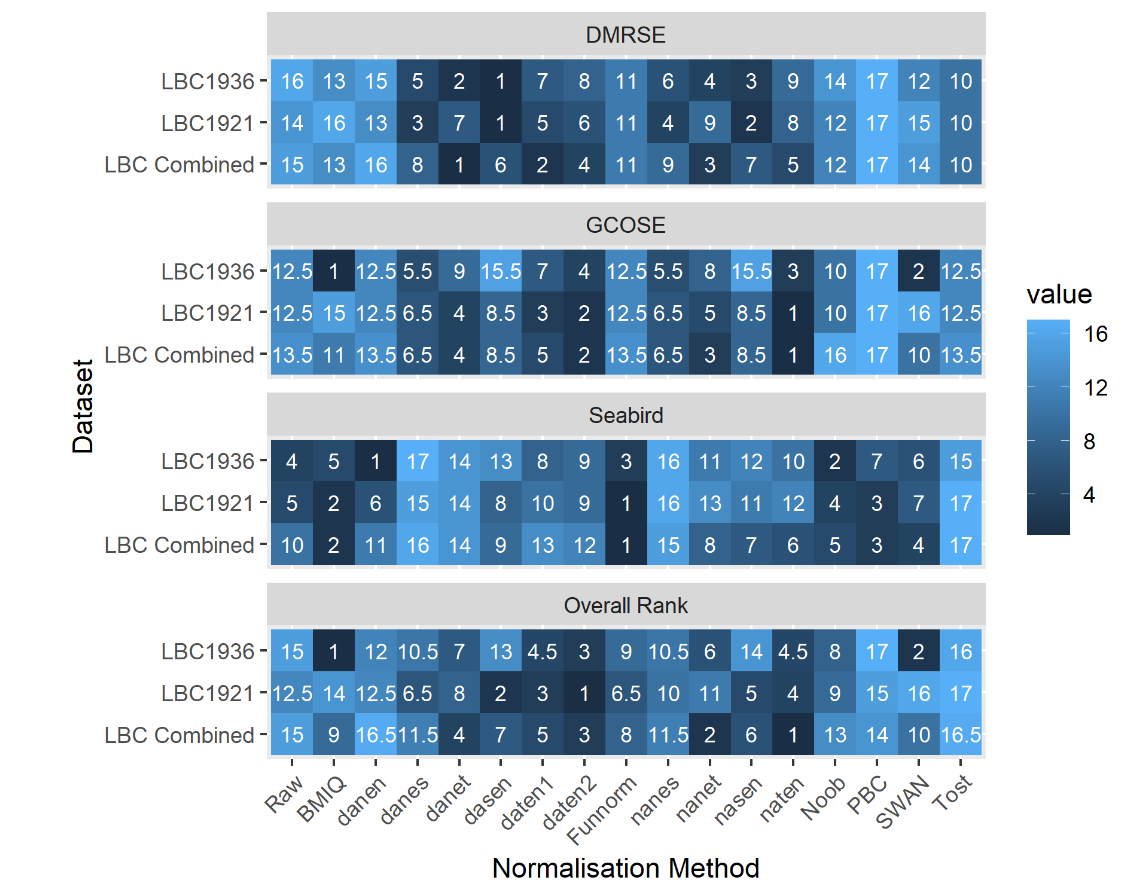


**Fig S2:** Individual scores for the DMRSE, GCOSE and Seabird metrics across normalisation methods applied to LBC1921, LBC1936 and both cohorts combined.


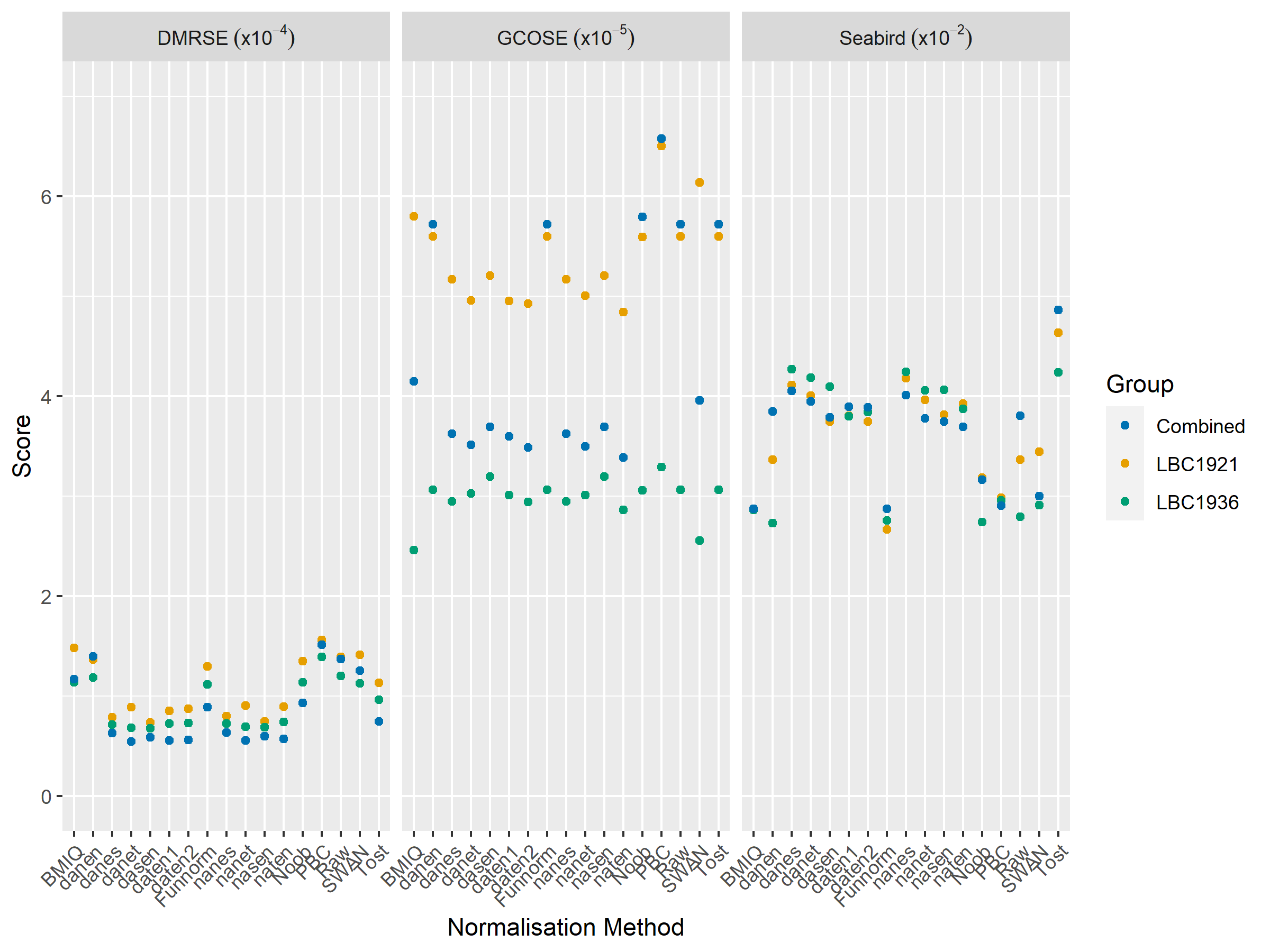


**Fig S3:** Density plot of BMI (kg/m^2^) in the Lothian Birth Cohort 1921, the Lothian Birth Cohort 1936, and Generation Scotland.


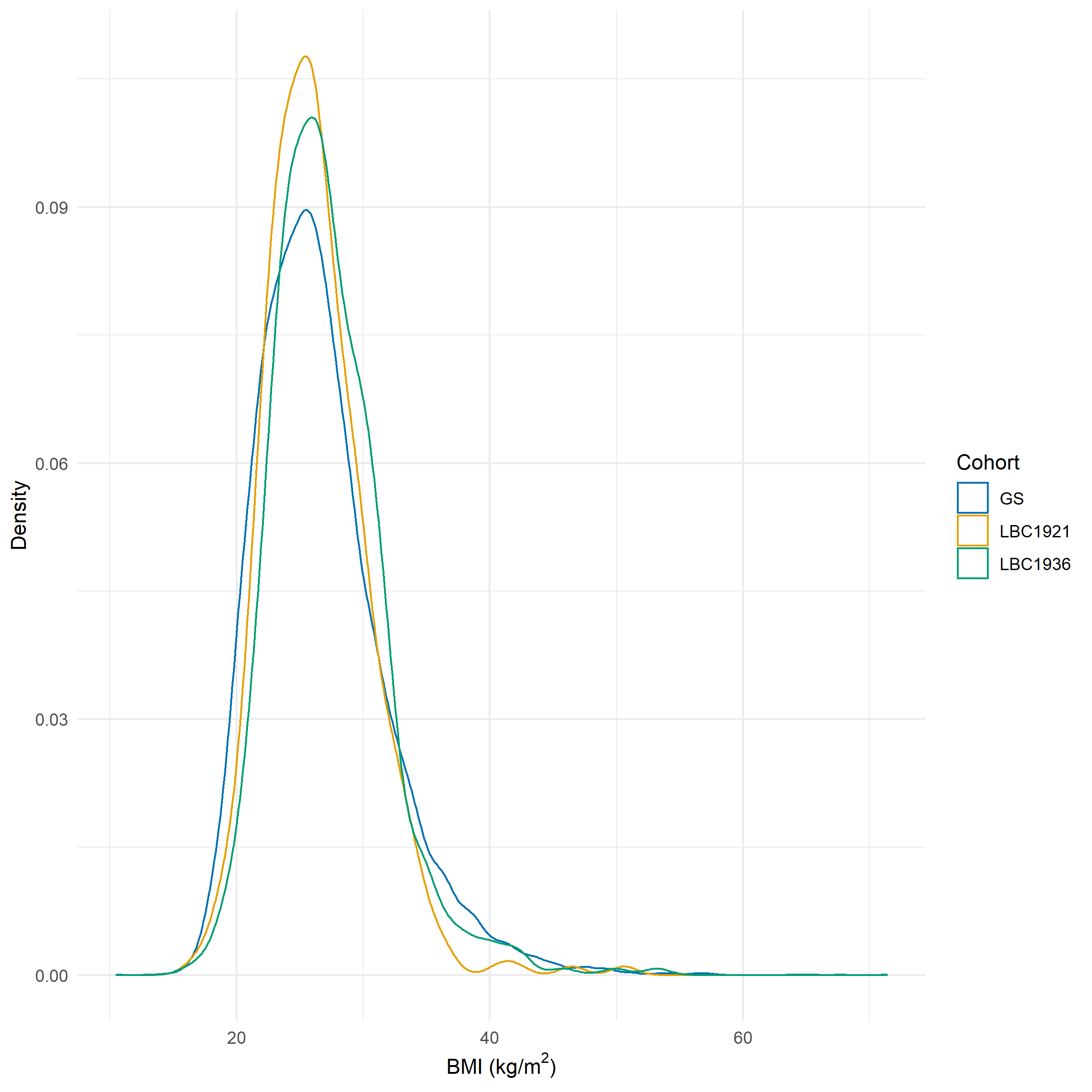


**Appendix 1:** Converting BMI EpiScores back to the original (BMI kg/m^2^) scale.

The following formula was used to rescale the BMI EpiScores:

$$x1=\mathrm{mean}_{\mathrm{lm}}+{{(SD}_{\mathrm{lm}} x BMI}_{\mathrm{test}})$$

$$x2=coef_{\mathrm{intercept}}+(coef_{\mathrm{age}} x \mathrm{age}_{\mathrm{test}})+(coef_{\mathrm{sex}} x \mathrm{sex}_{\mathrm{test}})$$

$$rescale=exp(x1+x2)$$

Where *lm* is the residual from the model log(BMI) ~ age + sex in the training set (Generation Scotland), *test* is the corresponding test sample (LBC1921, LBC1936 or Combined), and *coef* is the set of coefficients from the model *lm*.
