# Supplementary material for: Integration of datasets for individual prediction of DNA methylation-based biomarkers
Source: Genome Biol. 2023 Dec 5;24:278. doi: 10.1186/s13059-023-03114-5 (PMC10696831; doi:10.1186/s13059-023-03114-5)
Supplement: Supplementary file 2 — Additional file 2: Table S1. Correlations between measured BMI vs BMI EpiScore and age vs age EpiScore in LBC1921, LBC1936 and LBC1921 + LBC1936 (Combined) for every normalisation method. Table S2. WateRmelon normalisation method descriptions. [file 13059_2023_3114_MOESM2_ESM.docx]

**Table S1:** Correlations between measured BMI vs BMI EpiScore and age vs age EpiScore in LBC1921, LBC1936 and LBC1921 + LBC1936 (Combined) for every normalisation method. The largest value in each column is highlighted in bold.

|  | **BMI** | | | **Age** | | |
| --- | --- | --- | --- | --- | --- | --- |
|  | **Pearson Correlation** | | | | | |
| **Normalisation Method** | **LBC1921** | **LBC1936** | **Combined** | **LBC1921** | **LBC1936** | **Combined** |
| BMIQ | 0.321 | 0.478 | 0.427 | 0.085 | 0.052 | 0.080 |
| danen | 0.337 | 0.481 | 0.436 | 0.100 | 0.084 | 0.114 |
| danes | 0.353 | 0.469 | 0.426 | 0.144 | 0.072 | 0.115 |
| danet | 0.339 | 0.469 | 0.433 | 0.134 | 0.014 | 0.102 |
| dasen | 0.352 | 0.472 | 0.428 | 0.127 | 0.076 | 0.112 |
| daten1 | 0.347 | 0.473 | 0.431 | 0.133 | 0.024 | 0.103 |
| daten2 | 0.342 | 0.47 | 0.432 | 0.131 | 0.021 | 0.101 |
| PBC | 0.332 | 0.477 | 0.432 | 0.071 | 0.037 | 0.070 |
| Funnorm | 0.31 | 0.482 | 0.412 | 0.130 | **0.095** | **0.118** |
| nanes | **0.357** | 0.468 | 0.428 | 0.141 | 0.073 | 0.114 |
| nanet | 0.349 | 0.464 | 0.431 | 0.134 | 0.026 | 0.101 |
| nasen | 0.356 | 0.472 | 0.431 | 0.124 | 0.078 | 0.111 |
| naten | 0.351 | 0.468 | 0.433 | 0.131 | 0.034 | 0.103 |
| Noob | 0.311 | 0.478 | 0.423 | 0.124 | 0.087 | 0.116 |
| SWAN | 0.337 | **0.494** | **0.445** | 0.081 | 0.082 | 0.079 |
| Tost | 0.311 | 0.45 | 0.407 | **0.150** | 0.004 | 0.094 |

**Table S2**. WateRmelon normalisation method descriptions.

|  | Description of Normalisation Methods | | |
| --- | --- | --- | --- |
| Normalisation | Background  Adjustment | Between-Array normalisation | Dye-Bias Correction |
| naten | None | Type I/II together | None |
| nanet | None | None | Type I/II together |
| nanes | None | None | Type I/II separately |
| nasen | None | Type I/II separately | None |
| danet | Adjusted | None | Type I/II together |
| danes | Adjusted | None | Type I/II separately |
| dasen | Adjusted | Type I/II separately | None |
