# Supplementary material for: Integration of datasets for individual prediction of DNA methylation-based biomarkers
Source: Genome Biol. 2023 Dec 5;24:278. doi: 10.1186/s13059-023-03114-5 (PMC10696831; doi:10.1186/s13059-023-03114-5)
Supplement: Supplementary file 4 — Additional file 4: Review history. [file 13059_2023_3114_MOESM4_ESM.docx]

Review History

**First round of review**

**Reviewer 1**

**Were you able to assess all statistics in the manuscript, including the appropriateness of statistical tests used?**

No, I do not feel adequately qualified to assess the statistics.

**Comments to author:**

This study describes the comparison of different normalisation techniques for DNA methylation microarray data, to identify the best method for integrating datasets for the calculation of epigenetic scores. This is a useful and important endeavour to enable the application of these scores in clinical or commercial settings. However, before publication there are a few issues that need to be addressed.

1. The authors state that by selecting the most robust normalisation method they will be able to project a 'new individual onto a reference panel'. However all the comparisons and results presented here are performed on entire datasets (n=387 and 498) - i.e. projecting one dataset onto another, not an individual onto a dataset. The 'nanet' and 'danet' method that the study finds to be the most robust are both between-array normalising methods, so the data normalisation depends on the signal intensities from the entire cohort. If the authors want to claim that these methods are robust for integrating individual samples, then they also need to demonstrate this by conducting comparisons of single samples versus those single samples normalised together with a reference cohort. (So far they have only shown how well two whole datasets can be combined).

2. Figure 2 is not the best way to present this data as the reader can not easily compare normalisation methods, except by referring to Supp Table 1. Parallel bar charts such as in fig 3 would be a much more suitable way of displaying the data. Related to this figure do the authors have an explanation as to why the episcore predictor would be more accurate in LBC1936 compared to LBC1921? Also discussion is needed as to how the correlation between the episcore and actual BMI compares to the correlations found in their previous work. If the correlation is low between actual BMI and predicted BMI for each dataset, are they the right datasets on which to compare normalisation methods for robustness?

3. Can the authors discuss more about the mean difference in Figure 4 and their conversion to kg/m2. For example is a difference of 0.01 kg/m2 small or large? or an acceptable degree of error?

**Reviewer 2**

**Were you able to assess all statistics in the manuscript, including the appropriateness of statistical tests used?**

Yes, and I have assessed the statistics in my report.

**Comments to author:**

The authors build on Pidsley et al 2013, by doing a very similar survey of performance across a range of normalisation or preprocessing methods for Illumina DNA methylation arrays.

This work goes beyond Pidsley by using a much larger dataset and partially addressing the question of what to do when combining datasets or applying predictors from one dataset on another and test some newer methods.

While this kind of methods analysis is extremely worthwhile, and the work is of great interest to people in the field of DNAm phenotype prediction, the current study has some limitations which are not fully acknowledged. This is a problem in that it makes the conclusions potentially misleading.

One limitation (shared with the Pidsley paper) is that the data used for testing is very homogeneous, technically clean data that would largely work without normalisation. This is the quality of data that the authors are interested in using, but many readers would get different mileage when applying their conclusions to the wide range of data found in the wild. It makes it difficult to discriminate the performance of methods because there's not much to normalise away.

The authors derive a BMI predictor from Generation Scotland and use the correlation of the prediction with measured BMI as a performance metric. This is attractive in that it uses something that users of the data will be interested in, but may have a problem that could be leading the authors to the wrong conclusion. The LBC1921 and 1936 birth cohorts are quite comparable and the BMI and DNAm data are probably excellent, but any systematic error that happens to exaggerate the BMI score would cause normalisation methods that remove some of the the systematic error to perform "worse" by this metric. Given that this is what the authors in fact see, they should confront this possible explanation for their results.

They include in their analysis a number of 'within-array' methods which manipulate aspects of the intensity or beta value distribution of each sample, but don't do anything about minimising systematic error. These perform similarly to 'raw' and apparently better than methods which do normalisation as we normally understand it. As the authors acknowledge, the differences they are looking at are small and they highlight (probably chance) best performance by SWAN, a recommendation that is very unlikely to be of general application.

**Authors Response**

**Point-by-point responses to the reviewers’ comments****:**

**Reviewer #1:**

This study describes the comparison of different normalisation techniques for DNA methylation microarray data, to identify the best method for integrating datasets for the calculation of epigenetic scores. This is a useful and important endeavour to enable the application of these scores in clinical or commercial settings. However, before publication there are a few issues that need to be addressed.

*Response: We thank the reviewer for their positive feedback and importantly, for raising several excellent points that we have addressed below.*

1. The authors state that by selecting the most robust normalisation method they will be able to project a 'new individual onto a reference panel'. However all the comparisons and results presented here are performed on entire datasets (n=387 and 498) - i.e. projecting one dataset onto another, not an individual onto a dataset. The 'nanet' and 'danet' method that the study finds to be the most robust are both between-array normalising methods, so the data normalisation depends on the signal intensities from the entire cohort. If the authors want to claim that these methods are robust for integrating individual samples, then they also need to demonstrate this by conducting comparisons of single samples versus those single samples normalised together with a reference cohort. (So far they have only shown how well two whole datasets can be combined).

***Response:*** *The reviewer highlights an important point. We have amended the manuscript to reflect the performance of these methods when combining at the dataset level, rather than the individual level (all edited text highlighted in yellow below). We also acknowledge this point in the discussion, highlighting that we would expect the majority of applications would involve combining datasets, rather than a single individual with a reference dataset, due to the cost and time associated with sample collection and data generation. By definition, if we were to consider new individuals one-at-a-time then only within array normalisation methods would be applicable. To highlight the spread of the error across individuals in the population, we have now added boxplots (median error and IQR) in Figure 3:*

*Abstract:*

*“Projecting a new ~~individual~~ dataset onto a reference panel”*

*Introduction:*

“*Understanding the impact of normalisation is vital if future studies are to integrate multiple methylation datasets ~~or project new individuals onto existing datasets~~.*”

“*Here, we apply various quantile normalisation methods to two independent cohorts of age-matched older adults (considered separately and jointly) to determine which approach performs best for the projection of ~~an individual or~~ a new set of individuals onto a reference dataset.*”

*Discussion:*

*“The removal of technical noise from DNAm datasets is essential for more reliable projections ~~predictions~~ of ~~a~~ new individuals onto a reference dataset.”*

*“When projecting EpiScores for a new ~~individual/~~cohort, ..”*

*Discussion:*

“*Furthermore, we focused on population average differences across the normalisation strategies, as opposed to variation at the individual level. By definition, if we were to consider new individuals one-at-a-time then only within array normalisation methods would be applicable.* *When projecting new DNAm data into an existing dataset, it is likely that any new samples will be analysed in experimental batches due to the costs and time associated with generating the data. Further research should investigate the potential consequences of different normalization strategies when incorporating data from individual samples into existing datasets.*”

Updated Figure 3

2. Figure 2 is not the best way to present this data as the reader can not easily compare normalisation methods, except by referring to Supp Table 1. Parallel bar charts such as in fig 3 would be a much more suitable way of displaying the data. Related to this figure do the authors have an explanation as to why the episcore predictor would be more accurate in LBC1936 compared to LBC1921? Also discussion is needed as to how the correlation between the episcore and actual BMI compares to the correlations found in their previous work. If the correlation is low between actual BMI and predicted BMI for each dataset, are they the right datasets on which to compare normalisation methods for robustness?

***Response:*** *We thank the reviewer for these suggestions. To improve readability we have replotted Figure 2 as point estimate correlations with 95% CIs. The difference in the BMI EpiScore performance between LBC1291 and LBC1936 is difficult to explain. Previously, we noted this in the discussion. We have now added some text (highlighted in yellow) to hypothesise why this might be.*

Updated Figure 2:

*“Its correlation with observed BMI differed substantially across the two LBC studies, despite minimal heterogeneity in age and background. We hypothesise that these differences may be a result of selection or survival bias. LBC1921 participants were required to be healthy upon recruitment at age 79 compared to LBC1936 volunteers, who were healthy upon recruitment at age 70 but had to survive until the fourth study wave (at age 79) to be considered in these analyses.”*

*The final point raised about the suitability of comparing normalisation methods for EpiScores that have a low/modest correlation with the outcome trait is an excellent one. We have added the following text to the discussion along with new analyses for a second complex trait, chronological age:*

*Discussion:*

*“The maximum effect size observed here (R2 = 24.5%) is larger than the previous best estimate (19.5%) trained in a smaller subset of Generation Scotland and tested in the same LBC1936 cohort [17]. This is a much larger effect size than we observed from a BMI polygenic risk score (R2 = 10.1%) [2] and is also comparable to the largest effect sizes previously observed for 109 protein EpiScores (see Figure 2 from [3]). Chronological age is the most widely reported trait with large EpiScore correlations (i.e. Epigenetic Clocks). However, while these clocks show high correlations across populations with wide-age ranges, they perform much less well in birth cohorts or studies with a narrow age range. Applying Horvath’s 2013 predictor [15] to our current datasets yielded a different order of ‘best’ normalisation methods, though nanet performed well across both traits (BMI and age). Considering both the trait as well as the normalisation method is therefore likely to be of importance when combining datasets.”*

Results:

“*Prediction and robustness for Epigenetic Age*

*To explore if the findings varied by the EpiScore being tested, we re-ran the analyses for Horvath’s 2013 predictor of chronological age [15]. Whereas epigenetic clocks typically show correlations above 0.9 in populations with wide age ranges, they are less robust within birth cohorts. Here, we observed correlations between 0.004 and 0.15 across the cohorts and normalisation methods. We also observed a different rank ordering of the median errors for the normalisation approaches. The nanet method performed consistently well across predicted age and BMI, whereas the daten1 method showed consistently poor performance across traits. Mixed performance was observed for other methods (e.g. danet).”*

3. Can the authors discuss more about the mean difference in Figure 4 and their conversion to kg/m2. For example is a difference of 0.01 kg/m2 small or large? or an acceptable degree of error?

***Response:*** *This is another excellent point. Quantifying an acceptable degree of error is likely to vary both by EpiScore and its application e.g., trying to select high risk individuals for a clinical trial or treatment based on an absolute cut-off. What one might consider to be more important is the preservation of rank order e.g., that a new individual doesn’t change their %ile place in the population distribution too much if their data are normalised separately or together with the reference population (assuming that the normalisation approach yields an EpiScore with high enough prediction accuracy. We have added the following text (highlighted in yellow) to the discussion to cover this:*

*Discussion:*

*“Ideally, studies would not have to re-normalise a dataset every time new volunteers or patients are entered, which is both time consuming and computationally expensive. Quantifying an acceptable median absolute difference between EpiScores generated from different normalisation approaches is likely to vary by both the EpiScore and its application. What might be more important is the preservation of rank order e.g., if this is used to help select individuals for a clinical trial. Ideally, a new individual wouldn’t change their percentile place in a population distribution if their data are normalised separately or together with the reference dataset.”*

**Reviewer #2:**
The authors build on Pidsley et al 2013, by doing a very similar survey of performance across a range of normalisation or preprocessing methods for Illumina DNA methylation arrays.

This work goes beyond Pidsley by using a much larger dataset and partially addressing the question of what to do when combining datasets or applying predictors from one dataset on another and test some newer methods.

While this kind of methods analysis is extremely worthwhile, and the work is of great interest to people in the field of DNAm phenotype prediction, the current study has some limitations which are not fully acknowledged.  This is a problem in that it makes the conclusions potentially misleading.

*Response: We thank the reviewer for their positive remarks. We agree that some of the limitations need to be better highlighted. We have done this and re-worked our conclusions accordingly. Our responses to each specific point are presented below.*

One limitation (shared with the Pidsley paper) is that the data used for testing is very homogeneous, technically clean data that would largely work without normalisation.  This is the quality of data that the authors are interested in using, but many readers would get different mileage when applying their conclusions to the wide range of data found in the wild.  It makes it difficult to discriminate the performance of methods because there's not much to normalise away.

***Response:*** *The reviewer raises a very important point here. We have added the following text (new text in yellow) to discuss this in more detail:*

*Discussion:*

*“Differences are also likely to exist when EpiScores are applied to more diverse populations (e.g., wider age ranges, different social backgrounds and ancestries), though the tightly-matched age range across both test cohorts reported here is valuable in that cross-cohort comparisons were not confounded by age differences. The controlled environment in which samples were collected (in a clinic, according to a protocol) and processed (in the same research laboratory) may also be a limitation in that much of the heterogeneity associated with data collection and processing of clinical samples will not be present in our data. Therefore, prior to establishing a “best practice” model, further testing in diverse samples are required. EpiScore predictors for more complex traits or diseases risk scores could also be trained [2].”*

The authors derive a BMI predictor from Generation Scotland and use the correlation of the prediction with measured BMI as a performance metric.  This is attractive in that it uses something that users of the data will be interested in, but may have a problem that could be leading the authors to the wrong conclusion.  The LBC1921 and 1936 birth cohorts are quite comparable and the BMI and DNAm data are probably excellent, but any systematic error that happens to exaggerate the BMI score would cause normalisation methods that remove some of the the systematic error to perform "worse" by this metric.  Given that this is what the authors in fact see, they should confront this possible explanation for their results.

***Response:*** *We agree with the reviewer that systematic errors may result in different conclusions being made. In an effort to address this, we have extended the EpiScore analysis to age, which has no systematic error in its measurement and also shows large correlations with its EpiScore at the population level.*

*We have added the following section to the results section to report these new findings:*

Results:

“*Prediction and robustness for Epigenetic Age*

*To explore if the findings varied by the EpiScore being tested, we re-ran the analyses for Horvath’s 2013 predictor of chronological age [13]. Whereas epigenetic clocks typically show correlations above 0.9 in populations with wide age ranges, they are less robust within birth cohorts. Here, we observed correlations between 0.004 and 0.15 across the cohorts and normalisation methods. We also observed a different rank ordering of the median errors for the normalisation approaches. The nanet method performed consistently well across predicted age and BMI, whereas the daten1 method showed consistently poor performance across traits. Mixed performance was observed for other methods (e.g. danet). ”*

They include in their analysis a number of 'within-array' methods which manipulate aspects of the intensity or beta value distribution of each sample, but don't do anything about minimising systematic error. These perform similarly to 'raw' and apparently better than methods which do normalisation as we normally understand it.  As the authors acknowledge, the differences they are looking at are small and they highlight (probably chance) best performance by SWAN, a recommendation that is very unlikely to be of general application.

***Response:*** *This is a valid point and we have addressed it in the discussion section (new text highlighted in yellow):*

*Discussion:*

*“While within-array methods result in a zero mean absolute difference, they also guarantee that no batch-level effects are taken into account. Specifically, they do not attempt to minimise systematic errors that may have taken place during data collection and processing. It is therefore possibly a chance finding (or a result of relatively homogeneous input data) that SWAN yielded the best BMI EpiScore correlation with measured BMI. Perhaps reflecting this, another within-array method, Noob, yielded the second-lowest correlation between BMI and the EpiScore in LBC1921 and the jointly normalised cohorts.”*

**Second round of review**

**Reviewer 2**

The authors have made a good effort to satisfy reviewer comments on the original manuscript. They have added a second predictor (the Horvath 2013 clock) to help address one of my previous points. In principle this strengthens the paper but the authors should change the statistic they are using because it muddies the waters. The ms says "Whereas epigenetic clocks typically show correlations above 0.9 [...] they are less robust within birth cohorts". This is not about the performance of the clocks, but rather about the unsuitability of correlation as a measure here because the birth cohorts have very little variance in age and predicted age.

**Authors Response**

**Point-by-point responses to the reviewers’ comments:**

Reviewer #2:

The authors have made a good effort to satisfy reviewer comments on the original manuscript. They have added a second predictor (the Horvath 2013 clock) to help address one of my previous points. In principle this strengthens the paper but the authors should change the statistic they are using because it muddies the waters. The ms says "Whereas epigenetic clocks typically show correlations above 0.9 [...] they are less robust within birth cohorts". This is not about the performance of the clocks, but rather about the unsuitability of correlation as a measure here because the birth cohorts have very little variance in age and predicted age.

*Response: We have amended the text to reflect that correlation may not be appropriate with invariant data, such as age among birth cohorts:

‘Whereas epigenetic clocks typically show correlations above 0.9 in populations with wide age ranges, this metric is less sensitive within narrow age ranges (e.g. birth cohorts).’*
